# Erratum: Zwischen maximalem Einsatz und Herausforderungen: Interdisziplinäre Netzwerke in der ambulanten Palliativversorgung im Modell Westfalen-Lippe – Ergebnisse einer qualitativen Studie

**DOI:** 10.1055/a-2945-0077

**Published:** 2026-09-10

**Authors:** Anastasia Suslow, Chantal Giehl, Nino Chikhradze, Horst Christian Vollmar, Ina Otte

**Affiliations:** 1 Abteilung für Allgemeinmedizin (AM RUB) 9142 Ruhr University BochumBochumGermany; 2 Klinik für Altersmedizin und Frührehabilitation 169393 Universitätsklinik Marien Hospital HerneHerneNRWGermany; 3 Abteilung für VersorgungsforschungRuhr-Universität Bochum, Ruhr-Universität BochumBochum44801Germany; 4 Abteilung für Allgemeinmedizin (AM RUB) 9142 Ruhr-Universitat BochumBochumNRWGermany; 5 Abteilung für Versorgungsforschung, Institut für Diversitätsmedizin 9142 Ruhr-Universität BochumBochumNordrhein-WestfalenGermany



Im oben genannten Artikel wurde der Name des Autors Horst Christian Vollmar in den Metadaten fehlerhaft ausgezeichnet. Korrekt ist: Vorname: Horst Mittelname: Christian Nachname: Vollmar Die Korrektur wurde in der Onlineversion des Artikels ausgeführt am: 28. August, 2026.

